# Long-Term Effects of Chronic Buspirone during Adolescence Reduce the Adverse Influences of Neonatal Inflammatory Pain and Stress on Adaptive Behavior in Adult Male Rats

**DOI:** 10.3389/fnbeh.2017.00011

**Published:** 2017-01-26

**Authors:** Irina P. Butkevich, Viktor A. Mikhailenko, Elena A. Vershinina, Anna M. Aloisi, Gordon A. Barr

**Affiliations:** ^1^Laboratory of Ontogenesis of the Nervous System, I.P. Pavlov Institute of Physiology, Russian Academy of SciencesSt. Petersburg, Russia; ^2^Department of Information Technologies and Mathematical Modeling, I.P. Pavlov Institute of Physiology, Russian Academy of SciencesSt. Petersburg, Russia; ^3^Department of Medicine, Surgery and Neuroscience, University of SienaSiena, Italy; ^4^Department of Anesthesiology and Critical Care Medicine, The Children’s Hospital of Philadelphia and the Perelman School of Medicine at the University of PennsylvaniaPhiladelphia, PA, USA

**Keywords:** neonatal pain/stress, maternal deprivation, adaptive behavior, buspirone, adult rats

## Abstract

Neonatal pain and stress induce long-term changes in pain sensitivity and behavior. Previously we found alterations in pain sensitivity in adolescent rats exposed to early-life adverse events. We tested whether these alterations have long-lasting effects and if those effects can be improved by the 5-hydroxytryptamine 1A (5-HT1A) receptor agonist buspirone injected chronically during the adolescent period. This study investigates: (1) effects of inflammatory pain (the injection of formalin into the pad of a hind paw) or stress (short maternal deprivation-isolation, MI), or their combination in 1–2-day-old rats on the adult basal pain, formalin-induced pain, anxiety and depression; (2) effects of adolescent buspirone in adult rats that experienced similar early-life insults. Changes in nociceptive thresholds were evaluated using the hot plate (HP) and formalin tests; levels of anxiety and depression were assessed with the elevated plus maze and forced swim tests respectively. Both neonatal painful and stressful treatments induced long-term alterations in the forced swim test. Other changes in adult behavioral responses were dependent on the type of neonatal treatment. There was a notable lack of long-term effects of the combination of early inflammatory pain and stress of MI on the pain responses, anxiety levels or on the effects of adolescent buspirone. This study provides the first evidence that chronic injection of buspirone in adolescent rats alters antinociceptive and anxiolytic effects limited to adult rats that showed behavioral alterations induced by early-life adverse treatments. These data highlight the role of 5-HT1A receptors in long-term effects of neonatal inflammatory pain and stress of short MI on adaptive behavior and possibility of correction of the pain and psychoemotional behavior that were altered by adverse pain/stress intervention using buspirone during critical adolescent period.

## Introduction

Clinical and laboratory findings indicate that early-life painful and stressful events result in unfavorable neurodevelopmental outcomes: adult animals demonstrate increased sensitivity to clinical and experimental stimuli (Anand et al., 1999; Ren et al., 2004; Grunau, 2013; Walker et al., 2016), altered function of the hypothalamo-pituitary-adrenal axis (HPA) and behavioral changes (Anseloni et al., 2005; Rincón-Cortés and Sullivan, 2014; Brummelte et al., 2015; Valeri et al., 2015; Victoria and Murphy, 2016). The neonatal period is a time of neuropil expansion, synapse formation and elevated brain plasticity, during which the developing nervous system is characterized by heightened sensitivity to changes in sensory experience (Rice and Barone, 2000). Adverse consequences of pain and stress during this critical period are crucial for brain development and neurogenesis, the generation of new neurons and their interrelationships, which determine normal function of the adult brain (Leslie et al., 2011; Korosi et al., 2012).

Pain in newborn children in the neonatal intensive care unit is accompanied by the stress of separation from the mother (maternal deprivation). Using a rodent model of early-life pain, we previously found disrupted adaptive behavior in adolescent rats exposed as neonates to inflammatory pain or stress of maternal deprivation, with the consequences of early adverse insults dependent on the type and the age when they occur (Butkevich et al., 2015). In the present study, we use 1–2-day-old rats whose brain development is similar to that of a 24-week premature human infant (Anand et al., 1999; Schwaller and Fitzgerald, 2014). Premature children experience significant pain from repeated therapeutic procedures while hospitalized in the neonatal intensive care unit (Gibbins et al., 2015). The mechanisms of long-term influences of early-life pain and stress on nociception and stress responses are not known and are the subject of much clinical and basic research (Schwaller and Fitzgerald, 2014; Brummelte et al., 2015; Victoria and Murphy, 2016; Walker et al., 2016).

The need to understand the risks of untreated pain in neonates and of intensive analgesic practice using opiates, which induce long-term unfavorable effects on neuronal development, has stimulated further research aimed at preventing the consequences of adverse early-life events, including both non-pharmacological (McNair et al., 2013) and pharmacological (Walker and Yaksh, 2012) treatments of early-life pain. Our study on the use of the 5-hydroxytryptamine 1A (5-HT1A) receptor agonist buspirone for prevention of disorders of painful and psycho-emotional behaviors in prenatally stressed rats (Butkevich I. P. et al., 2011; Butkevich I. et al., 2011) suggested that buspirone can reduce or eliminate the adverse effects of neonatal pain and stress in the adolescent brain and improve resilience of nociceptive functions in adulthood.

Buspirone is in clinical use as an anxiolytic that does not cause dependance. It is also effective in the treatment of depression, often in combination with other antidepressants due to its molecular properties (Albert and François, 2010; Albert and Fiori, 2014). Buspirone is a full agonist of presynaptic 5-HT1A receptors (autoreceptors) located on the axons, soma and dendrites of serotonergic neurons in the dorsal and median raphe nuclei; activation of 5-HT1A receptors inhibits raphe firing rates and the release of serotonin (5-HT) through a negative feedback mechanism (Hannon and Hoyer, 2008). Buspirone is a partial agonist of postsynaptic 5-HT1A receptors (heteroreceptors) that present in the hippocampus, hypothalamus, thalamus and amygdala, where these receptors are located on GABAergic interneurons. At these postsynaptic sites, 5-HT1A receptor activation can have an inhibitory effect on the neuronal activity induced by various neurotransmitters. The responses of pre- and post-synaptic 5-HT1A receptors to treatment with antidepressants have not been adequately addressed because of difficulties in separating effects on autoreceptors from effects on heteroreceptors. There are different views on the involvement of pre- and postsynaptic 5-HT1A receptors in the anxiolytic and antidepressant effects of buspirone (Albert and Fiori, 2014). Studies on the use of buspirone for pain relief are limited and the results are inconsistent (Pavlaković et al., 2009; Chang et al., 2015).

Our hypothesis is that adolescent treatment with buspirone will normalize the altered reactivity of the adult nociceptive system that had experienced adverse events in the newborn period. This hypothesis is supported by the finding of a period in adolescence with heightened neurodevelopmental plasticity, which provides opportunities to re-direct programming stemming from neonatal life experiences (McCormick and Green, 2013). The involvement of 5-HT1A receptor agonists in neuroprotective effects is also known (Kline et al., 2004), as is the ability of buspirone to increase hippocampal neurogenesis in adult rodents (Mori et al., 2014). New data on the anti-inflammatory properties of buspirone (Sharifi et al., 2015) suggest its involvement in normalization of the level of inflammatory cytokines previously altered by adverse experiences; this contributes to restoration of afferent-efferent connections of the raphe nuclei with the prefrontal cortex and spinal cord which are involved in integration of the antinociceptive and psycho-emotional systems.

The present study consists of two series of experiments. The aim of the first was to investigate effects neonatal inflammation-induced pain and/or stress of maternal deprivation-isolation (MI) on adult basal pain, pain responses to ongoing similar inflammation, and the levels of anxiety and depression in the adult rats. The aim of the second series was to evaluate long-term effects of chronic injections of buspirone during adolescence on the indices of adaptive behaviors in adult rats that had experienced neonatal insults identical to those in the first series.

## Materials and Methods

### Animals

Female (*n* = 43) and male (*n* = 23) Wistar rats were obtained from the vivarium of the I.P. Pavlov Institute of Physiology. The animals were mated and a vaginal smear was examined to verify insemination (Butkevich I. et al., 2011). The days of insemination and delivery were designated as gestational day 0 and postnatal day 0, respectively. Pregnant rats were initially housed four per cage and then individually after the 17th day of pregnancy. All animals were maintained under standard conditions (12 h light, 12 h dark, lights on at 08:00) in standard plastic rat cages with food and water available *ad libitum*. The birth of litters was checked twice daily at 9 AM and 6 PM. On postnatal day one (P1) the litters were culled to eigth pups per dam. The present study was approved by the Animal Care and Use Committee of the I.P. Pavlov Institute of Physiology, Russian Academy of Sciences (St. Petersburg, Russia). Animal care was conducted in accordance with the EC Council Directive of November 1986 (86/609/EEC).

### Experimental Design

When the rats reached adult age (90 day-old male rats) the effects of neonatal noxious insults on indices of adaptive behaviors (basal pain in the hot plate (HP) test, inflammatory pain response in the formalin test, psycho-emotional behaviors in the elevated plus maze and forced swim tests) were studied (Series I). In Series I, male rats on P1 and on P2 (P1, 2) were exposed to the following treatments: a single injection of formalin (FOR group) or saline (control to formalin), after which the pups were immediately returned to their dams and siblings (*n* = 11, *n* = 12 respectively); a single injection of FOR or saline and immediate isolation from the dams and siblings for 60 min (MI, “psychogenic stressor” (FOR followed by MI group), saline followed by MI group, *n* = 9, *n* = 9, respectively). The formalin concentration (2.5%), the volume of FOR or saline (0.5 μl), the site of injection (a single subcutaneous injection into the pad of the left hind paw) and the “psychogenic stressor” (each pup placed singly in a small cage in a thermostat at 30°C for 60 min) were similar to those in our previous work (Butkevich et al., 2015). The injection of FOR (but not that of saline) induced flexing and shaking of the injected paw, i.e., formalin-induced pain behavior (licking behavior is immature in neonatal pups). For all but three litters we tested a maximum of one littermate from a single litter for each condition; however, in those three litters there were 2–3 rats from each litter (three subjects twice and two subjects once). Where there was the more than a single rat from the same litter, the data from the multiple pups were averaged to provide a single value for that litter. Thus the unit of analysis was litter, a single pup in most litters and the average of multiple pups in three litters. In preliminary experiments we found no differences in any of the tested parameters between adult rats treated with saline on P1, 2 and naïve animals (not exposed to any stimuli on P1, 2). Therefore, naïve rats were considered as controls (CTL). Weaning was carried out on P25, and the male rats were housed in standard cages (four per cage) until testing on P90.

In series II and III we examined the effects of the saline control (Series II) or buspirone (Series III) during adolescence on the similar indices like in the Series I. In the Series III possible favorable effects of adolescent buspirone (BUS) on the similar behaviors altered by neonatal treatments were evaluated. In these both series, rats were exposed to the same treatments like in Series I on P1, 2 and then were treated in the following experimental conditions from P25 to P39: chronic injections (daily injections) of saline (SAL, control for buspirone) or buspirone (buspirone hydrochloride, BUS, Sigma, 3.5 mg/kg, 1 ml i.p. at 9 AM). Buspirone exhibits its medicinal effect if used for a long time. Thus the following groups of rats were formed: FOR, *n* = 8 and *n* = 11; MI, *n* = 8 and *n* = 10; FOR followed by MI, *n* = 9 and *n* = 11; CTL, *n* = 12 and *n* = 12, for SAL (Series II) and BUS (Series III) respectively. The buspirone dose and the duration of the treatment were from our previous study (Butkevich I. P. et al., 2011; Butkevich I. et al., 2011).

### Adult Behavioral Tests

On P90 the rats were examined in a battery of tests conducted in the following order: assessment of basal pain in the HP test, of inflammatory pain in the formalin test, of anxiety and depression in the elevated plus maze and forced swim test respectively. The formalin test occurred the day after the HP test, the following tests (the elevated plus maze and the forced swim test) were performed with the interval in 3 days. The experiments were conducted between 09:00 AM and 2:00 PM. Behavior in all the tests was observed visually and recorded by video camera. Observers were blind to the early-life treatment of the animals. Each rat was taken randomly from the cage and after the HP test was labeled with a picric acid solution. Rats were returned to their home cage at the end of each test.

### Hot Plate (HP) Test

Baseline nociceptive sensitivity was assessed using the HP test. The apparatus consists of a 25 cm × 25 cm metal HP surface set at 51°C, a Plexiglas cage that fits over the HP and a foot-switch timer. A rat was taken randomly from the nest and placed on the HP. Latency to the first lifting the paw is usually considered an indication of pain threshold. We terminate the test if there is no response during 30 s. The latency was averaged from three trials with 10 min intervals between each trial. The testing apparatus was thoroughly cleaned between trials.

### Formalin Test

The formalin test is widely used to assess intensity of inflammatory pain response to formalin injection. The response (flexing and licking behaviors) consists of the acute and tonic phases (Barrot, 2012). The test lasted 60 min. The first acute phase lasts for about 5 min after the formalin injection and is considered to arise from direct activation of myelinated and unmyelinated nociceptive afferent fibers. The second tonic phase (about 50 min) is considered to result from changes in central nervous system (CNS) function induced by neural activity generated during the first phase and from the developing inflammation caused by the formalin during the second phase (Coderre et al., 1993). We measured licking, mediated at the supraspinal level, in response to formalin injection (2.5%, 50 μl into the pad of the left hind paw) during the acute and tonic phases (Butkevich I. et al., 2011) using a computer program specially created for our experiments that allows recording, quantification and analysis of the formalin-induced pain behaviors.

### Elevated Plus Maze (EPM)

The elevated plus maze is a widely used measure of anxiety in rodents (Wall and Messier, 2001). The standard elevated plus maze (two opposing open arms, 50 cm × 10 cm and two other arms of the same size having 50 cm high transparent Plexiglas walls; the maze is raised 100 cm above the floor) was used to evaluate the anxiety level. During the 5-min test we recorded the time spent in the open arms (s) and the number of unprotected head dips which negatively correlate with the level of anxiety.

### Forced Swim Test (Modification of the Porsolt Test) (Porsolt et al., 1977)

The forced swim test is one of the most widely used measures of an animal’s ability to cope with stress. Each rat was individually placed into the glass cylinder filled with water (diameter 25 cm, height 60 cm, 24 ± 1°C). The time of immobility (the rat only makes movements necessary to keep the head above the water) was recorded during the 5-min test. This parameter, characterizing the level of depression, is widely considered a negative index of the animal’s ability to cope with stress.

### Statistical Analyses

For all analyses, we averaged the data from littermates (where they were 2 or 3 from a litter) to create a single data point per litter. Thus the unit of analysis was the litter. All data are presented as mean ± SEM. We analyzed the data with either a 3 × 2 × 2 ANOVA (the HP test, the forced swim test) or 3 × 2 × 2 MANOVA (the formalin test, elevated plus maze) to test the effects of the different experimental conditions (Series I without SAL and BUS; Series II with SAL; Series III with BUS), Pain (CTL, FOR) and Stress (CTL, MI) on the following indices: basal pain, licking duration (two phases), anxiety (number of unprotected head dips and time spent in the open arms), depression (time of immobility). Multiple comparisons and simple effect tests were performed with Bonferroni’s test. The partial eta square (*η*^2^) was used to estimate effect size. *P* < 0.05 was considered statistically significant. Data analysis was carried out with the SPSS Inc. software.

## Results

### Hot Plate (HP) Test

**Figure 1:**
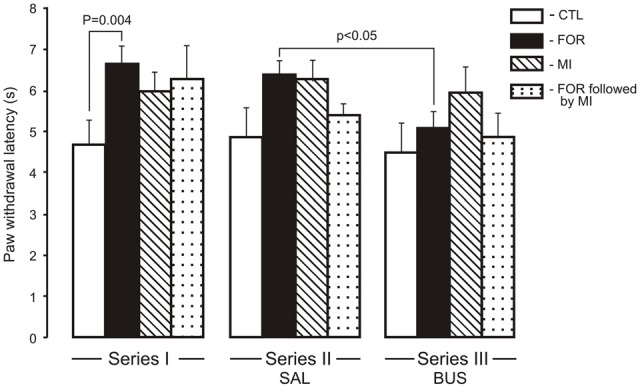
**Effect of adolescent buspirone on paw withdrawal latency in the hot plate (HP) test in adult rats exposed to different treatments (FOR, MI, FOR followed by MI) on P1, 2**. FOR, inflammatory pain induced by injection of formalin (FOR group); MI, maternal deprivation-isolation (MI group); FOR followed by MI, combination of inflammatory pain and maternal deprivation-isolation (FOR followed by MI group). Series I: without adolescent saline (SAL) and adolescent buspirone (BUS); Series II: with adolescent SAL; Series III: with adolescent BUS. CTL, control group (naïve rats). SAL, adolescent chronic injection of saline from P25 to P39 to rats in each group (CTL, FOR, MI, FOR followed by MI); BUS, adolescent chronic injection of buspirone from P25 to P39 to rats in each group (CTL, FOR, MI, FOR followed by MI). Data are mean ± SEM.

For the HP latency there were main effects of Pain and Stress (*F*_(1,110)_ = 6.93, *p* = 0.010, *η*^2^ = 0.059 and *F*_(1,110)_ = 6.52, *p* = 0.012, *η*^2^ = 0.056, respectively) and a significant interaction between Pain and Stress (*F*_(1,110)_ = 9.7, *p* = 0.001, *η*^2^ = 0.081). The neonatal inflammatory treatment produced bilateral basal thermal hypoalgesia in the adult rats exposed to FOR on P1, 2. *Post hoc* testing showed a significant increase of HP latency in adult rats exposed to FOR on P1, 2 with respect to CTL (naïve rats; *p* = 0.004) in the Series I. There were no significant differences in the HP latency between CTL and other groups (FOR, MI and FOR followed by MI) in Series II and Series III. Chronic injection of adolescent buspirone (Series III) in the rats exposed to FOR on P1, 2 decreased the HP latency as compared to the HP latency in FOR rats with adolescent SAL (Series II; *p* < 0.05). Adolescent BUS did not affect on paw withdrawal latency in CTL rats (Figure 1).

### Formalin Test

**Figure 2:**
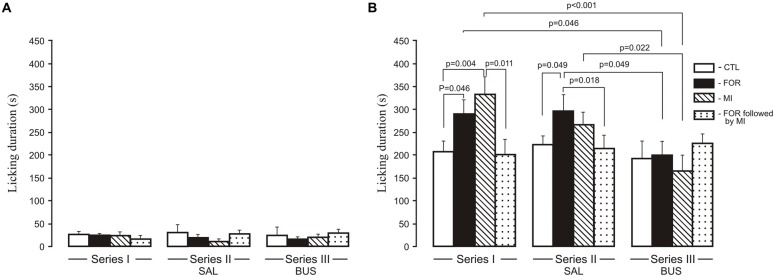
**Effect of adolescent buspirone on licking duration in the formalin test during the first phase (A)** and the second phase **(B)** in adult rats exposed to different treatments (FOR, MI, FOR followed by MI) on P1, 2. FOR, inflammatory pain induced by injection of formalin (FOR group); MI, maternal deprivation-isolation (MI group); FOR followed by MI, combination of inflammatory pain and maternal deprivation-isolation (FOR followed by MI group). Series I: without adolescent saline (SAL) and adolescent buspirone (BUS); Series II: with adolescent SAL; Series III: with adolescent BUS. CTL, control group (naïve rats). SAL, adolescent chronic injection of saline from P25 to P39 to rats in each group (CTL, FOR, MI, FOR followed by MI); BUS, adolescent chronic injection of buspirone from P25 to P39 to rats in each group (CTL, FOR, MI, FOR followed by MI). Data are mean ± SEM.

For licking duration there was a significant multivariate main effect of Experimental Condition (Wilks’ *λ* = 0.923, *F*_(4,214)_ = 2.2, *p* = 0.07, *η*^2^ = 0.039) and a significant interaction between Pain and Stress (Wilks’ *λ* = 0.867, *F*_(2,107)_ = 8.18, *p* < 0.001, *η*^2^ = 0.133). In addition, the interaction between the Experimental Condition, Pain and Stress was also significant (Wilks’ *λ* = 0.885, *F*_(4,214)_ = 3.63, *p* = 0.011, *η*^2^ = 0.059). Given the significance of the overall test, the univariate main effects were examined. There were significant univariate main effect of Experimental Condition for licking duration for the second phase of the formalin test only (*F*_(2,108)_ = 4.0, *p* = 0.020, *η*^2^ = 0.07), and a significant interaction between Pain and Stress (*F*_(1,108)_ = 8.97, *p* = 0.003, *η*^2^ = 0.077) and between the Experimental Condition, Pain and Stress (*F*_(2,108)_ = 5.73, *p* = 0.004, *η*^2^ = 0.096). There were no significant influences of early-life treatments (FOR, MI, FOR followed by MI) on licking duration in the first phase of the formalin test for any Series, or any effect of adolescent BUS (Series III) on licking duration as compared to the adolescent SAL (Series II) in any groups (Figure 2A). In the second phase of the formalin test, in Series I, *post hoc* testing showed an increase in licking duration during the second phase in adult rats exposed to FOR or MI on P1, 2 (*p* = 0.046 and *p* = 0.004, respectively) as compared to CTL (naïve rats; Figure 2B). Licking duration was not altered in adult rats that had experienced the combination of pain and stress on P1, 2; their licking duration was significantly lower than in the MI group (*p* = 0.011; Figure 2B). In Series II, chronic injection of SAL during the adolescence increased licking duration in rats exposed to FOR on P1, 2 as compared to CTL (*p* = 0.049), but was not changed in rats exposed to MI and to FOR followed by MI. Licking duration in the latter was significantly lower than in FOR animals (*p* = 0.018). In Series III, there were no significant differences in licking duration between CTL and other groups (FOR, MI and FOR followed by MI). Chronic injection of adolescent buspirone in the rats exposed to FOR as well as to MI on P1, 2 reduced licking duration as compared to the effect of chronic injection of adolescent SAL (Series II; *p* = 0.049 and *p* = 0.022 respectively) and compared to Series I in the rats of similar groups (*p* = 0.046 and *p* < 0.001 respectively). There was no effect of adolescent buspirone on licking duration in rats in the group of FOR followed by MI (Figure 2B).

### Elevated Plus Maze (EPM)

**Figure 3:**
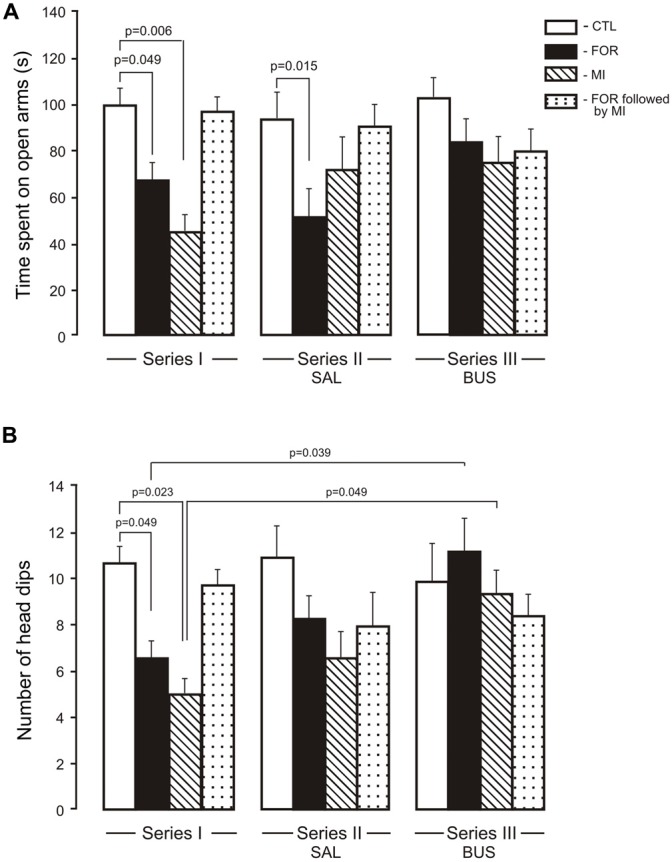
**Effect of adolescent buspirone on the indices of anxiety (A**, time spent on open arms, **B**, the number of head dips) in adult rats exposed to different treatments (FOR, MI, FOR followed by MI) on P1, 2. FOR, inflammatory pain induced by injection of formalin (FOR group); MI, maternal deprivation-isolation (MI group); FOR followed by MI, combination of inflammatory pain and maternal deprivation-isolation (FOR followed by MI group). Series I: without adolescent saline (SAL) and adolescent buspirone (BUS); Series II: with adolescent SAL; Series III: with adolescent BUS. CTL, control group (naïve rats). SAL, adolescent chronic injection of saline from P25 to P39 to rats in each group (CTL, FOR, MI, FOR followed by MI); BUS, adolescent chronic injection of buspirone from P25 to P39 to rats in each group (CTL, FOR, MI, FOR followed by MI). Data are mean ± SEM.

The MANOVA for anxiety revealed a significant multivariate main effect of Stress (Wilks’ *λ* = 0.949, *F*_(2,109)_ = 2.95, *p* = 0.049, *η*^2^ = 0.051), and a significant interaction between Experimental Condition and Stress (Wilks’ *λ* = 0.905, *F*_(4,218)_ = 2.8, *p* = 0.027, *η*^2^ = 0.049) and between Pain and Stress (Wilks’ *λ* = 0.948, *F*_(2,109)_ = 2.99, *p* = 0.049, *η*^2^ = 0.052). Given the significance of the overall test, the univariate main effects were examined. There were significant univariate main effects of Stress for Elevated plus maze (EPM) for the number of head dips (*F*_(1,110)_ = 5.7, *p* = 0.019, *η*^2^ = 0.049) and for an interaction between Experimental Condition, Pain and Stress (*F*_(2,110)_ = 4.10, *p* = 0.019, *η*^2^ = 0.069). For the time spent in the open arms there were significant interactions between Experimental Condition and Stress (*F*_(2,110)_ = 3.21, *p* = 0.044, *η*^2^ = 0.055), between Pain and Stress (*F*_(1,110)_ = 5.8, *p* = 0.017, *η*^2^ = 0.05) and between the Experimental Condition, Pain and Stress (*F*_(2,110)_ = 2.7, *p* = 0.054, *η*^2^ = 0.047). In Series I, adult rats exposed to FOR or MI on P1, 2 showed a decrease in the time spent in the open arms (*p* = 0.049 and *p* = 0.006, respectively) and in the number of unprotected head dips (*p* = 0.049 and *p* = 0.023) as compared to CTL. The combination of FOR and MI on P1, 2 did not change these indices relative to CTL (Figures 3A,B). In Series II, chronic injection of SAL during the adolescence decreased the time spent in the open arms in rats exposed to FOR and to MI on P1, 2 as compared to CTL (*p* = 0.015 and *p* = 0.06 respectively), but not in rats exposed to FOR followed by MI; there were also significant differences in the number of head dips between CTL and other groups (FOR, MI and FOR followed by MI). In Series III, there were no differences between CTL and other groups (FOR, MI and FOR followed by MI) in either the time spent in the open arms or in the number of unprotected head dips. In both indices of anxiety there were no significant differences between the effects of adolescent BUS and SAL in any groups of rats. In Series III, chronic injection of adolescent buspirone in the rats exposed to FOR and to MI on P1, 2 increased the number of head dips as compared to this index in the similar groups in Series I (*p* = 0.039 and *p* = 0.049 respectively) and normalized the number of head dips (Figure 3B).

### Forced Swim Test

**Figure 4:**
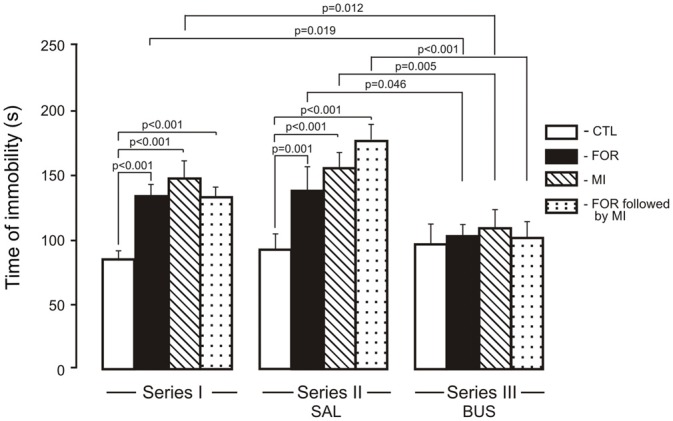
**Effect of adolescent buspirone on the immobility time in adult rats exposed to different treatments (FOR, MI, FOR followed by MI) on P1, 2.** FOR, inflammatory pain induced by injection of formalin (FOR group); MI, maternal deprivation-isolation (MI group); FOR followed by MI, combination of inflammatory pain and maternal deprivation-isolation (FOR followed by MI group). Series I: without adolescent saline (SAL) and adolescent buspirone (BUS); Series II: with adolescent SAL; Series III: with adolescent BUS. CTL, control group (naïve rats). SAL, adolescent chronic injection of saline from P25 to P39 to rats in each group (CTL, FOR, MI, FOR followed by MI); BUS, adolescent chronic injection of buspirone from P25 to P39 to rats in each group (CTL, FOR, MI, FOR followed by MI). Data are mean ± SEM.

The ANOVA for immobility revealed significant main effects of Experimental Condition, Pain and Stress (*F*_(2,110)_ = 12.1, *p* < 0.001, *η*^2^ = 0.180, *F*_(1,110)_ = 9.88, *p* = 0.002, *η*^2^ = 0.082 and *F*_(1,110)_ = 27.7, *p* < 0.001, *η*^2^ = 0.201, respectively) and significant interactions between Experimental Condition and Stress and between Pain and Stress (*F*_(1,110)_ = 3.56, *p* = 0.032, *η*^2^ = 0.061, *F*_(1,110)_ = 13.99, *p* < 0.001, *η*^2^ = 0.113 respectively). In Series I, adult rats exposed on P1, 2 to FOR, MI and FOR followed by MI showed an increase in the time of immobility (*p* < 0.001 in all cases) as compared to CTL (Figure 4). In Series II, chronic injection of SAL during the adolescence increased the time of immobility in rats exposed on P1, 2 to FOR, MI and FOR followed by MI as compared to CTL (*p* = 0.001, *p* < 0.001, *p* < 0.001, respectively). In Series III, there were no differences between CTL and other groups (FOR, MI and FOR followed by MI). In Series III, chronic injection of adolescent buspirone in the rats exposed on P1, 2 to FOR, MI and FOR followed by MI decreased the time of immobility as compared to these indices in the similar groups in Series I (*p* = 0.019, *p* = 0.012, *p* = 0.055 respectively) and in Series II (*p* = 0.046, *p* = 0.005, *p* < 0.001 respectively) and normalized the time of immobility in all the groups (Figure 4).

## Discussion

The present study demonstrates that repeated inflammatory pain and stress in neonatal rats induce long-term alterations in adaptive behaviors depending on the type of treatment. In the adult rats exposed to inflammatory pain on P1, 2 there was a general decreased pain sensitivity to thermal stimulation in the HP test. In the formalin test, we found hyperalgesia in adult rats that had experienced inflammatory pain or the “psychogenic stress” of MI as neonates. The lack of long-term effects of the combination of early pain and MI on pain sensitivity and emotionality is of particular interest. The level of anxiety in the adult rats depended on the type of early treatment; all rats showed impaired stress coping ability irrespective of the type of neonatal treatment. Our results provide the first evidence that chronic injections of buspirone during the critical adolescent period normalize basal pain and pain sensitivity in the formalin test and the indices of psycho-emotional behavior in the adult rats that demonstrated behavioral alterations induced by early-life adverse treatments.

The data from the HP test in adult rats exposed to formalin pain on P1, 2 are in agreement with literature data on hypoalgesia in adult rats after inflammatory pain evoked during the neonatal period by an injection of carrageenan (CAR) and complete Freund’s adjuvant (CFA), agents with a more prolonged action than formalin (Lidov, 2002). However, the data on influences of early-life formalin-induced pain are inconsistent. For instance, early-life pain caused by formalin injection in rodents led to hyperalgesia in later life (Mohamad et al., 2011) or had no effect (Negrigo et al., 2011) in the HP test. The discrepancy between the results with neonatal formalin pain can be explained by the different concentration of formalin and the number of injections, as well as by the age of the neonatal rats (the former authors used 5%, 10 μl, once a day for the first three postnatal days, while the latter used two unilateral plantar injections of 4% formalin, 10 μl with an 1 h interval between them on P1).

It was suggested that hypoalgesia results from upregulated endogenous opioid tone in brain areas mediating pain (Laprairie and Murphy, 2009; Victoria and Murphy, 2016). The opioidergic system, as well as the monoaminergic system, is involved in the descending inhibitory modulation of the nociceptive signals and these systems can act together and independently (Hurley et al., 2003). Hypoalgesia, which presents regardless of the segmental level, is believed to be associated with more widespread mechanisms beyond the somatotopically organized nociceptive pathways of the dorsal horn, also involving the brainstem descending pain control system and the HPA system (Anseloni et al., 2005; Schwaller and Fitzgerald, 2014). However, the impact of early injury on this temporally regulated descending control of the developing and reorganizing dorsal horn requires further investigation (Beggs, 2015; Walker et al., 2016). For the HPA axis responses, a similar bi-directional effect was suggested (Victoria et al., 2013).

In the formalin test, rats exposed to neonatal inflammatory pain and immediately returned to the dam and the rats exposed to MI before being returned to the dam displayed heightened hyperalgesia as adults. Obviously, favorable factors like reunion with the dam and siblings and the odor of the nest were insufficient to suppress the consequences of differing adverse events. An elevated level of corticosterone in response to adverse experiences in newborn rat pups can influence the development of the stress system and later lead to changes in the response to formalin-induced pain. Indeed, literature data show that early-life stress in rats decreases the nociceptive threshold and enhances inflammatory mediator-induced hyperalgesia in adults, and inflammatory pain experienced on the day of birth alters adult responses to stress- and pain-provoking stimuli and deregulates the HPA axis (Victoria and Murphy, 2016). Since we used short-term MI, we cannot compare our results with the available literature data since only the effects of much more prolonged maternal deprivation on different types of behavior or HPA axis development have been studied (Nishi et al., 2014). Therefore short (60 min) isolation from the dam and siblings on P1 and repeatedly on P2 increases inflammatory pain when the rats reach the adulthood.

We obtained unexpected results regarding the pain response of adult rats exposed to the combination of pain and MI. In these adult rats, the formalin-induced pain response did not differ from the control pain response; moreover, it was significantly lower than the pain response in adult rats with the other neonatal treatments. It is also surprising that the combination of pain and stress on P1, 2, when the HPA axis still actively responds to irritants, failed to alter the pain sensitivity in the adult rats. Our results suggest that neonatal injury-induced changes evoked within the CNS by the interaction between the HPA axis and the nociceptive system in the neonatal rats exposed to FOR followed by MI contribute to the response to re-inflammation when these animals are adults. The functional activity of the HPA axis in 1–2-day-old rat pups is still high, since the so-called stress hyporesponsive period (SHRP; Levine, 1994) begins on postnatal day 4, when rat pups have low basal corticosterone levels and the corticosterone response to stressors is blunted. Indeed, we previously demonstrated that neonatal male rats exposed to formalin followed by maternal isolation had increased plasma corticosterone levels as the formalin-induced pain progressed during a 1-h period; the higher corticosterone levels persisted the next day (Butkevich et al., 2013). Other authors reported that plasma corticosterone levels were elevated in P1 rat pups after pain evoked by CFA injection and remained high for at least 1 week post-inflammatory injury (Victoria et al., 2014).

Pain and stress in newborn rat pups may alter characteristics of the nociceptive system and HPA axis development. In newborn rats, the nociceptive system, in which 5-HT1A receptors are important for the structural and functional development of neural circuits, shows high sensitivity to painful stimuli (Barr, 1998; Fitzgerald, 2005; Barr and Hunter, 2014). This is due to several major factors such as immaturity of C-fibers and the dominant role of low-threshold A-fibers in nociception (Beggs et al., 2002), transient excitatory gamma-aminobutyric acid (GABA) function at the spinal and supraspinal levels (Hatfield, 2014), and immaturity of the descending monoaminergic system (Butkevich I. P. et al., 2011). Early-life adverse effects at the peripheral and central levels alter the structural and functional development of the afferent fibers and their relationships (Beggs, 2015).

Anxiety-like behavior, measured as the time spent in the open arms of the EPM and the number of unprotected head dipping, was significantly increased in adult rats that had experienced inflammatory pain or MI as neonates. Interestingly, the same rats showed heightened hyperalgesia in the formalin test. When comparing the EPM and formalin test results, it is reasonable to suggest that the heightened level of anxiety and increased pain sensitivity shown by the various indices of adaptive behavior in the adult rats exposed to both early-life pain and stress are interrelated. The literature reports multi-faceted effects of early-life pain on anxiety-like behavior. For instance, newborn rats injected with CAR in the first postnatal week displayed reduced anxiety-like behavior as adults, thought to be due to increased activity of the opioid system (Anseloni et al., 2005; Victoria et al., 2014). In contrast, early-life hind-paw inflammation with formalin failed to alter anxiety in this test in male rats (Negrigo et al., 2011). Importantly, the markedly increased level of anxiety in our rats with early-life pain or stress contrasted with the lack of long-term changes in the anxiety indices in the rats with the combination of early-life pain and stress. The latter did not show also any changes in the formalin-induced pain response. These interesting data on unusual consequences of the combination of early-life pain and stress on the development of adaptive behavior and nociception are worthy of particular attention. In the forced swim test, all infant treatments altered the depression phenotype. However other authors found that CAR-induced acute peripheral inflammation on P3 significantly decreased adult depression-related behaviors in the forced swim test (Anseloni et al., 2005). Differences in the experimental designs may contribute to the discrepancies between our results and the results of these authors.

Of particular interest is the behavior of the adult rats with the combination of early-life pain and stress. The behaviors in the HP test, formalin test and EPM in the rats of this group were unchanged, whereas the index of depression was significantly increased. This replicates our prior work showing that the combination of neonatal pain and stress in conditions of re-inflammation had no effect in adolescent rats (Butkevich et al., 2015). The means, by which there was a lower pain response in adult animals with greater overall neonatal adversity, are unknown but it is an important question in understanding the long-term effects of early pain and stress on adaptive behavior. Although definite mechanisms of adverse influences in early life on nociceptive pathways are not known, they could be mediated by the HPA axis, which affects the brainstem descending pain control systems and thus influences neurophysiological mechanisms underlying pain perception (Schwaller and Fitzgerald, 2014).

We also provide the first evidence that chronic injection of buspirone in adolescent rats exposed to early-life peripheral inflammatory pain or short-lasting MI normalized pain sensitivity and the level of depression and improved the level of anxiety when the rats were adults. Importantly, buspirone had antinociceptive, antidepressive and anxiolytic effects only in the adult rats that showed long-term behavioral alterations induced by early-life adverse injury. In adult rats with the combination of early-life pain and stress, buspirone had an effect only in the forced swim test, during which these rats showed heightened depression without buspirone injections, but not in the other tests, in which behavior was unchanged. There are clinical observations that buspirone affects patients with symptoms of anxiety and depression but not healthy patients. Our data on the effects of buspirone on both inflammatory pain and psychoemotional behavior in adult rats suggest that because buspirone is a full agonist of presynaptic 5-HT1A receptors and a partial agonist of postsynaptic 5-HT1A receptors (Albert and François, 2010; Albert and Fiori, 2014), it decreases pain sensitivity via 5-HT1A receptors in the descending monoaminergic inhibitory system (Butkevich I. P. et al., 2011). The 5-HT1A receptor plays an important role in neuronal development and plasticity (Whitaker-Azmitia et al., 1996). It is the main receptor in autoregulation of the brain 5-HT system (Samuels et al., 2016). In the spinal cord and brain, the 5-HT1A receptor is involved in nociceptive mechanisms (Colpaert, 2006) and mediates an inhibitory effect of the descending serotonergic system on inflammatory pain behavior (Millan, 1999). 5-HT1A receptors are implicated also in mood disorders (anxiety, depression; Popova and Naumenko, 2013). The other possibility is that buspirone, acting via 5-HT1A receptors during the adolescent period, can eliminate abnormalities in the CNS evoked by impairment of close interactions between the 5-HTergic system and the HPA axis. The 5-HTergic system and the HPA axis act jointly especially in brain regions involved in the neurobiological substrate of stressful and painful responses, such as the hippocampus and hypothalamus (Belay et al., 2011). Our previous data indicate that prenatal buspirone increases the plasma level of corticosterone in prenatally stressed rats during formalin-induced pain (Butkevich et al., 2013). Buspirone in early adolescent period activating the HPA axis via 5-HT1A receptors could lead to facilitation of some adaptive mechanisms disturbed by early adverse insults. The adolescent period is characterized with heightened neurodevelopmental plasticity, which provides opportunities to re-direct programming stemming from neonatal life experiences (McCormick and Green, 2013).

Previously we found in 25-day-old rats morphological abnormalities in the nucleus raphe magnus following neonatal inflammatory pain that heightened hyperalgesia in the formalin test; structural characteristics were improved by chronic injections of buspirone and pain sensitivity was normalized when the rats reached late adolescence (Butkevich et al., 2015; Droblenkov et al., 2015). This nucleus is involved in the descending serotonergic system, which modulates nociceptive signals at the spinal cord dorsal horns and has neuroanatomical connections with the prefrontal cortex (Bernasconi et al., 2015) involved in psycho-emotional behavior. Morphofunctional alterations in the cortex and hippocampus were found in adult rodents that had suffered early repeated pain injuries (Malheiros et al., 2014). At present we cannot know which type of 5-HT1A receptors is involved in effects of buspirone. There are different views on the involvement of pre- and postsynaptic 5-HT1A receptors in the anxiolytic and antidepressant effects of buspirone. A recent study using a genetic system to independently decrease levels of the 5-HT1A auto- and heteroreceptor population (Richardson-Jones et al., 2011) demonstrated that 5-HT1A autoreceptors affect anxiety-like behavior while 5-HT1A heteroreceptors affect behavioral despair responses. Buspirone as an antidepressant drug may increase 5-HT-ergic signaling at the postsynaptic receptor either through desensitization of the somatodendritic autoreceptors or through enhanced activation of G proteins at the postsynaptic 5-HT1A receptors.

In summary, our studies using the model of early-life pain/stress in P1, 2 rats for investigation of long-term effects of adverse injuries and adolescent buspirone injections on adaptive behavior in the adult male rats have demonstrated that alterations in the behavioral responses are differently influenced by the type of injury. Of particular interest is the lack of long-term effects of the combination of early pain and stress of short MI on basal pain, formalin-induced pain responses and anxiety. We provide the first evidence that chronic injection of buspirone during the critical adolescent period induces antinociceptive, antidepressive and anxiolytic effects only in those adult rats that showed behavioral alterations induced by early-life adverse experiences. The data highlight the role of 5-HT1A receptors in long-term effects of neonatal inflammatory pain and stress of short MI on adaptive behavior and indicate possibility of correction of the pain and psychoemotional behavior altered by adverse pain/stress intervention using buspirone during critical adolescent period. Further studies should be devoted to studying this problem in female rats with the aim to find sexual dimorphism in effects of adolescent buspirone on long-term influence of neonatal pain and stress injuries.

## Author Contributions

IPB and VAM: experimental design. IPB, VAM and EAV: collection of data, conduction of statistical analyses. IPB, VAM, EAV, AMA and GAB: interpretation and analysis of data, participated in the drafting and revising of the manuscript, reviewed and approved the final submitted manuscript.

## Financial Disclosures

The material is original research, has not been previously published and has not been submitted for publication elsewhere.

## Conflict of Interest Statement

The authors declare that the research was conducted in the absence of any commercial or financial relationships that could be construed as a potential conflict of interest.
